# Effect of Patient-Controlled Epidural Analgesia (PCEA) Based on ERAS on Postoperative Recovery of Patients Undergoing Gynecological Laparoscopic Surgery

**DOI:** 10.1155/2022/6458525

**Published:** 2022-03-21

**Authors:** Tianli Zhu, Weiming Lu, Wenjuan Wang, Li Zhou, Wen Yan

**Affiliations:** ^1^Department of Obstetrics Second Ward, Xinhua Hospital Affiliated to Shanghai Jiaotong University School of Medicine, Shanghai, China; ^2^Department of Obstetric One Ward, Xinhua Hospital Affiliated to Shanghai Jiaotong University School of Medicine, Shanghai, China

## Abstract

**Objective:**

To explore the effect of patient-controlled epidural analgesia (PCEA) based on enhanced recovery after surgery (ERAS) on the postoperative recovery of patients undergoing gynecological laparoscopic surgery.

**Methods:**

Between January 2019 and December 2020, 90 patients scheduled for gynecological laparoscopic surgery and assessed for eligibility were recruited and randomly assigned at a ratio of 1 : 1 to receive either conventional analgesic management (regular group) or PCEA based on ERAS (ERAS group). Comparisons of postoperative rehabilitation indicators, visual analogue scale (VAS) score, self-care ability, complications, and nursing satisfaction were conducted between the two groups.

**Results:**

The ERAS group had significantly shorter first exhaust time (FET), first defecation time (FDT), out-of-bed activity time (OAT), and length of stay (LOS) versus the regular group (*P* < 0.05). The VAS scores were significantly decreased after treatment, with lower results observed in the ERAS group (*P* < 0.05). The level of self-responsibility, self-concept, self-care skills, and health knowledge increased significantly in both groups after the intervention, and the ERAS group showed significantly higher results than the regular group (*P* < 0.05). The total incidence of complications in the ERAS group was significantly lower than that in the regular group (*P* < 0.05). Eligible patients given PCEA based on ERAS were associated with a higher nursing satisfaction (97.78%) versus conventional analgesic management (82.22%) (*P* < 0.05).

**Conclusion:**

The application of ERAS for postoperative PCEA management in gynecological laparoscopy provides promising results by effectively enhancing the quality of surgery and promoting rapid postoperative recovery, with a good safety profile.

## 1. Introduction

The common gynecological diseases that need surgical treatment include uterine fibroids, endometriosis, benign ovarian tumor, tubal pregnancy, pelvic organ prolapse, and gynecological malignant tumors. With the continuous development and promotion of lumpectomy technology, laparoscopy has been widely used in gynecologic surgery. Research has shown significantly alleviated incisional pain after gynecologic laparoscopy versus open surgery, but the incidence of postoperative pain remains as high as 79.2% [1]. In addition, women are more sensitive to pain than men, which is associated with a stronger pain response. Thus, perioperative analgesia is essential to improve postoperative quality of life of patients. Currently, configuration of pain pumps is conducted for pain relief according to different surgery types, the outcome of intraoperative anesthesia, and the patient's condition [2].

Patient-controlled analgesia allows the self-control analgesia by the patients, among which patient-controlled epidural analgesia (PCEA) is a common analgesic method with long-term experience over 20 years in obstetrics and gynecology [3]. PCEA can maintain the blood concentration of analgesic drugs close to the minimum effective concentration. It has a reliable analgesic effect and long duration, which is conducive to early out-of-bed activities and a low incidence of nausea and vomiting. However, in clinical practice, the insufficient knowledge, laxity, and inadequate details in the management of analgesia by medical staff may compromise the analgesic efficiency or even leads to ineffective analgesia [4], higher risks of surgical complications, and reduced patients' satisfaction, which hinders the postoperative recovery of the patients [5]. It was professor Kehlet of the University of Denmark who first proposed the ERAS [6]. Reports have found that many routine perioperative measures such as preoperative enemas and delayed postoperative feeding contribute negatively to postoperative recovery or even result in detrimental factors [7, 8]. With merits of prevention of preoperative enemas, excellent intraoperative anesthesic efficacy, early postoperative feeding, and reduction of unnecessary drain placement, ERAS is associated with less surgical stress, faster postoperative recovery, reduced postoperative complications, and fewer medical costs [9]. The basic principle of ERAS for anesthesia is to reduce postoperative discomfort and achieve rapid recovery. Short-acting anesthetics (such as sevoflurane, desflurane, and propofol) can be combined with short-acting opioid analgesics. In the disciplines of pancreas, gastrointestinal tract, colorectal, and urology, there have been formal ERAS-related guidelines based on evidence-based medicine [10–12]. ERAS has been effectively implemented in orthopedics, colorectal, gastrointestinal, breast surgery, and urology [13]. In 2016, some scholars [14] searched the literature on ERAS in gynecological surgery, reviewed other guidelines for ERAS in abdominal surgery, and proposed the application of ERAS in gynecology. However, the rehabilitation effect of ERAS protocol in patients under PCEA after gynecological laparoscopic surgery has been marginally explored. Accordingly, 90 patients scheduled for gynecological laparoscopic surgery were recruited to assess the effectiveness of PCEA based on ERAS on postoperative recovery of patients undergoing gynecological laparoscopic surgery. The results are as follows.

## 2. Materials and Methods

### 2.1. General Data

Between January 2019 and December 2020, 90 patients scheduled for gynecological laparoscopic surgery and assessed for eligibility were recruited as research subjects. This study was approved by the ethics committee of the hospital, and all the eligible patients provided written informed consent.

Inclusion criteria: (1) aged 18–60 years, (2) all patients were graded as I or II by the American Society of Anesthesiologists (ASA) classification, (3) patients with confirmed preoperative and postoperative diagnosis, (4) all patients underwent gynecologic laparoscopic surgery, and (5) general anesthesia combined with local or regional anesthesia was used for intraoperative anesthesia, and PCEA was used for postoperative analgesia.

Exclusion criteria: (1) patients with thrombosis before operation; (2) patients with blood coagulation dysfunction; (3) patients with obvious surgical contraindications such as intestinal obstruction, diarrhea, and infection; (4) patients with underlying diseases such as diabetes mellitus and hypertension; (5) patients with significant complications before and after surgery; (6) patients with severe disturbance of consciousness that prevented cooperation with this study, or (7) patients with malignant tumors.

The eligible patients were assigned at a ratio of 1 : 1 via the random number table method to either the ERAS group or the regular group. The baseline characteristics of the patients in the ERAS group (28 cases of ASA I and 17 cases of ASA II, mean age of [40.11 ± 3.71] years, mean BMI of [22.73 ± 2.81] kg/m^2^) were comparable with those of the patients in the regular group (27 cases of ASA I and 18 cases of ASA II, mean age of [40.75 ± 3.84] years, mean BMI of [22.37 ± 2.15] kg/m^2^) (*P* > 0.05).

### 2.2. Methods

#### 2.2.1. Regular Group

The regular group received conventional analgesic management. The patients were educated by the chief anesthesiologist and the responsible nurse of the ward about the use of the analgesic pump, and the above-mentioned PCEA analgesic pump was provided for pain relief after the operation. If analgesic complications or no significant pain relief were observed in 10 minutes after the use of the analgesic pump, appropriate analgesic measures were performed immediately.

#### 2.2.2. ERAS Group

The ERAS group received ERAS intervention. (1) Patients were lectured about gynecological surgery and epidural analgesia pumps and were educated about the procedure and its importance. (2) Health education before intervention: the patients were given health education on ERAS, including a detailed explanation of the ERAS concept and the stages of postoperative recovery before the relevant nursing measures and treatment. The patients were informed of the differences between preoperative and postoperative care measures, such as administration of glucose 2 hours before surgery without fasting and reduced duration of postoperative bed rest, which enhanced the patients' perioperative cooperation. (3) Perioperative psychological care: before surgery, the nursing staff actively communicated with the patients to assess their psychological conditions. Postoperatively, the patients were instructed to relax and informed of the surgical results. The patients' family members were instructed about home care for the patients to ensure sufficient family supports. (4) Postoperative pain care: the patients were explained the causes of postsurgical pain in plain language and given epidural analgesic pumps for pain relief after surgery. Relaxing and soothing music was provided to help them relax for better pain relief effects. (5) Complications prevention: the patients' conditions were assessed prior to the procedure to estimate the type, severity, and timing of potential complications for the formulation of contingency plans. In the case of complications, the corresponding plans were implemented immediately. (6) Postoperative early rehabilitation activities: after the surgery, pain assessments and matching analgesic measures were performed. The patients were instructed to do early rehabilitation exercises, with upper limb doing fist clenching—fist release—elbow flexion and extension—shoulder supination—shoulder broad joint lateral extension, for a total of 5 sets. The lower limbs were flexed and extended at the left and right knee joints and raised up, for a total of 5 sets. Both hands were placed on the abdomen to massage the abdomen clockwise five times. All exercises were performed once every two hours until exhaustion. (7) Postoperative dietary guidance: if the patients showed no adverse reactions such as nausea and vomiting two hours after surgery, they were allowed to drink 300 ml of carbohydrates. Liquid food was allowed within 4 hours after the surgery, and the diet was switched to a normal diet within 24 hours after the surgery. (8) Discharge guidance: the patients with good recovery were discharged as soon as possible and were instructed to exercise for better recovery of physical function.

### 2.3. Outcome Measures

(1) Postoperative rehabilitation indexes of the two groups were monitored and recorded, including first exhaust time (FET), first defecation time (FDT), out-of-bed activity time (OAT), and hospital length of stay (LOS). (2) The degree of pain: the visual analogue scale (VAS) [15] was used for scoring. In a 10 cm long ruler, every 1 cm of the ruler represented 1 point, totaling 10 points. 0 means no pain, and 10 means unbearable severe pain. The higher the score, the greater the pain. (3) Self-care ability: exercise of self-care agency (ESCA) was adopted. The scale consisted of 4 subitems of self-responsibility (8 items), self-concept (9 items), self-care skills (12 items), and health knowledge level (14 items), with a total of 43 valid items. Each item was divided into 5 levels, with 0 to 4 points. The higher the score, the better the self-care ability. (4) Complications include nausea and vomiting, bloating, and urinary tract infection. (5) Nursing satisfaction: nursing satisfaction was assessed using the self-made questionnaire of our hospital, with a total of 20 items, each with 5 points, for a total of 100 points. A score of ≥80 points was highly satisfied, 60∼80 points was satisfied, and <60 points was dissatisfied. Nursing satisfaction = (highly satisfied + satisfied)/total number of cases × 100%.

### 2.4. Statistical Processing

SPSS 23.0 software was used for data analyses. Quantitative data were expressed as ($\bar{x}\pm s$), and qualitative data were expressed as *n* (%). Unordered qualitative data, ordered qualitative data, quantitative data, and repeated measures data were analyzed by chi-square test, rank sum test, *t*-test, and ANOVA, respectively. Differences were considered statistically significant at a *P* value lower than 0.05.

## 3. Results and Discussion

### 3.1. Comparison of Postoperative Rehabilitation Indexes

In the regular group, the FET was 18.96 ± 2.97 h, the FDT was 28.74 ± 4.86 h, the OAH was 16.95 ± 3.41 h, and the LOS was 6.79 ± 1.08 h. While the above indicators in the ERAS group were 16.81 ± 2.74 h, 25.86 ± 4.71 h, 14.74 ± 2.82 h, and 4.84 ± 0.74 h, which were all significantly shorter than those of the regular group (all *P* < 0.05) (Table 1).

### 3.2. Comparison of VAS Scores and Self-Care Ability

In the regular group, the VAS score was 1.49 ± 0.17 before the intervention, 2.54 ± 0.59 at 6 h after the intervention, and 2.07 ± 0.67 at 12 h after intervention. In the ERAS group, the VAS scores at the three time points were 1.47 ± 0.11, 1.96 ± 0.41, and 2.07 ± 0.67. The VAS scores were significantly decreased after treatment in both groups, with lower results observed in the ERAS group (*P* < 0.05) (Table 2).

The self-care ability consisted of four domains of self-responsibility, self-concept, self-care skills, and health knowledge. Before the intervention, the scores of the four parts were comparable (all *P* > 0.05). After the intervention, all the scores were increased significantly in both groups, and the ERAS group showed significantly higher results than the regular group (all *P* < 0.05) (Table 3).

### 3.3. Comparison of Complications and Nursing Satisfaction

In the ERAS group, 1 case had postoperative nausea and vomiting, and 1 case had bloating. In the regular group, there were 5 cases of nausea and vomiting, 3 cases of bloating, and 2 cases of urinary tract infection. The total incidence of complications in the ERAS group was 4.44% and the regular group was 22.22%. The total incidence of complications in ERAS group was significantly lower than that in regular group (*P* < 0.05) (Table 4).

In the ERAS group, 33 cases were highly satisfied, 11 cases were satisfied, and 1 case was dissatisfied. In the regular group, 22 cases were highly satisfied, 15 cases were satisfied, and 8 cases were dissatisfied. PCEA based on ERAS was associated with a higher nursing satisfaction (97.78%) versus conventional analgesic management (82.22%) (*P* < 0.05) (Table 5).

## 4. Discussion

PCEA allows the self-control of the time and dose of anesthesia by patients and features an excellent therapeutic effect [16]. Nevertheless, individual differences prevent consistent analgesic effects in all patients. In addition, postoperative complications elicited by analgesics, including headache, bloating, skin itching, hypotension, nausea and vomiting, nerve root irritation infection, low back pain, and epidural catheter prolapse have also attracted much clinical attention [17].

ERAS is currently one of the main measures used in the perioperative management of surgical operations. ERAS-related gastrointestinal management studies have concluded that prolonged preoperative fasting, especially beyond 12 h, depletes patients' glycogen and energy reserves, increases surgical stress and trauma, and leads to insulin resistance and hyperglycemia, and the reduction of preoperative fasting duration is in no way to increase the incidence of complications such as reflux and misaspiration. Preoperative sedation should follow individualized selection to avoid rigid use of sedative drugs, especially routine application of long-acting sedatives within 12 h preoperatively. Preoperative application of short-acting sedatives has been reported to reduce patients' preoperative anxiety but lead to postoperative motor dysfunction, impairing their eating and walking performances, which suggests individualized selection for their use. In ERAS, the application of nitrous oxide should be avoided in gynecological laparoscopy due to its high incidence of postoperative nausea and vomiting. The use of EEG dual-frequency index to monitor the depth of anesthesia can reduce the dose of anesthetic drugs and contribute to rapid awakening. Postoperative nausea and vomiting are commonly seen after gynecological surgery. 70% of patients undergoing abdominal surgery experience postoperative nausea and vomiting, which aggravates the patient's pain and may prolong the hospital stay. It is suggested by ERAS that laparoscopic surgery and gynecological surgery are independent predictors of postoperative nausea and vomiting. It is recommended that patients undergoing abdominal surgery and applying emetogenic anesthetic or analgesic drugs routinely use antiemetic medications, avoid general anesthesia and the application of nitrous oxide and volatile anesthetics, reduce the dose of opioids and neostigmine, and use propofol to reduce the risk of postoperative nausea and vomiting. In addition, ERAS discourages the use of drains and catheters to reduce the potential for abdominal infections that are associated with painful discomfort and hinder out of bed activities.

In this study, the ERAS group adopted PCEA based on ERAS and achieved obtained significantly shorter FET, FDT, OAT, LOS, and lower VAS scores. On the basis of evidence-based medicine, it optimizes the patient's perioperative diet and pain management to reduce the psychological and physical impact from the operation, thereby mitigating postoperative adverse reactions, promoting the patient's rapid recovery, and shortening the LOS [18]. ERAS also enhances patient trust in medical staff and improves nursing satisfaction. The International ERAS Association made a standardized summary of ERAS in the field of gynecological oncology for the first time in 2016, including preoperative, intraoperative, and postoperative methods [19]. Preoperative preparation (preoperative consultation, preoperative education, skin disinfection, prevention of thrombosis, etc.) is the main measure to ensure the success of the operation and improve the patient's prognosis. The smooth implementation of the intraoperative ERAS protocols is conducive to maintaining the stability of the patient's vital signs and rapid postoperative recovery, including the optimization of anesthesia measures, intraoperative heat preservation, and fluid rehydration. Rehabilitation methods usually advocate bed rest after surgery, which, however, is usually associated with a significantly increased incidence of complications such as thrombosis.

The results of this study showed that the postoperative rehabilitation indicators of the ERAS group were better than the regular group. The ERAS group showed shorter FET, FDT, OAT, and LOS versus the regular group, which is in line with the core concept of ERAS to achieve rapid recovery of gastrointestinal function. It is attributed to the administration of a small amount of carbohydrate drinks 2 h before surgery to reduce postoperative gastrointestinal discomfort, reduce the occurrence of insulin resistance, and maintain negative nitrogen balance [20]. The results of the present study showed significantly decreased VAS scores after treatment, with lower results observed in the ERAS group (*P* < 0.05), indicating that the application of the ERAS to the management of PCEA after gynecological laparoscopy can effectively improve analgesic efficacy. It is presumably attributable to elevated levels of self-responsibility, self-concept, self-care skills, and health knowledge after the intervention [21]. Moreover, the lower incidence of complications and higher nursing satisfaction after the introduction of ERAS versus the conventional analgesic methods suggest a high safety profile and high application value of ERAS. The limitation of this study lies in the absence of exploring the effect of the ERAS concept in the perioperative period of patients undergoing gynecological laparoscopy, which will be investigated in future studies.

## 5. Conclusion

The application of ERAS for postoperative PCEA management in gynecological laparoscopy could promote the recovery of body function after surgery, reduce the incidence of postoperative complications, shorten the length of hospital stay, improve patients' medical experience, and enhance the quality of medical service. However, the small number of samples included in this study resulted in a certain bias of the results, and future studies will be conducted with a large sample size to obtain more reliable results.

## Figures and Tables

**Table 1:** Comparison of postoperative rehabilitation indexes between the two groups ($\bar{x}\pm s$).

Groups	*n*	FET (h)	FDT (h)	OAT (h)	LOS (h)
ERAS group	45	16.81 ± 2.74	25.86 ± 4.71	14.74 ± 2.82	4.84 ± 0.74
Regular group	45	18.96 ± 2.97	28.74 ± 4.86	16.95 ± 3.41	6.79 ± 1.08
*t*		3.569	2.855	3.350	9.992
*P*		<0.001	0.005	0.001	<0.001

**Table 2:** Comparison of VAS scores between the two groups ($\bar{x}\pm s$).

Groups	*n*	Before intervention	6 h after intervention	12 h after intervention
ERAS group	45	1.47 ± 0.11	1.96 ± 0.41	2.07 ± 0.67
Regular group	45	1.49 ± 0.17	2.54 ± 0.59	3.13 ± 0.81
F groups			106.723	
*P* groups			<0.001	
F time			74.603	
*P* time			<0.001	
F mutual			22.004	
*P* mutual			<0.001	

**Table 3:** Comparison of self-care ability between the two groups ($\bar{x}\pm s$).

Groups	*n*	Time	Self-responsibility	Self-concept	Self-care skills	Health knowledge levels
ERAS group	45	Before intervention	26.38 ± 2.53	28.93 ± 2.11	32.48 ± 3.76	34.57 ± 3.65
		After intervention	33.84 ± 3.96^*∗*^^#^	36.86 ± 3.18^*∗*^^#^	42.17 ± 4.24^*∗*^^#^	45.78 ± 4.24^*∗*^^#^
Regular group	45	Before intervention	26.14 ± 2.58	28.87 ± 2.24	32.53 ± 3.72	34.62 ± 3.62
		After intervention	30.46 ± 2.17^*∗*^	32.43 ± 3.39^*∗*^	37.35 ± 3.51^*∗*^	39.76 ± 4.01^*∗*^

*Note.* ^*∗*^, *P* < 0.05 in the comparison with before intervention in the same group; #*P* < 0.05 in the comparison with the regular group after intervention.

**Table 4:** Comparison of complications in the two groups (*n*, %).

Groups	*n*	Sick and vomit	Bloating	Urinary tract infection	Total incidence
ERAS group	45	1(2.22)	1 (2.22)	0	2 (4.44)
Regular group	45	5 (11.11)	3 (6.67)	2 (4.44)	10 (22.22)
*χ* ^2^					4.712
*P*					0.030

**Table 5:** Comparison of nursing satisfaction between the two groups (*n*, %).

Groups	*n*	Very satisfied	Satisfied	Not satisfied
ERAS group	45	33 (73.33)	11 (24.44)	1 (2.22)
Regular group	45	22 (48.89)	15 (33.33)	8 (17.78)
*Z*			−4.693	
*P*			<0.001	

## Data Availability

No data were used to support this study.
